# Multiparametric MR radiomics in brain glioma: models comparation to predict biomarker status

**DOI:** 10.1186/s12880-022-00865-8

**Published:** 2022-08-05

**Authors:** Jinlong He, Jialiang Ren, Guangming Niu, Aishi Liu, Qiong Wu, Shenghui Xie, Xueying Ma, Bo Li, Peng Wang, Jing Shen, Jianlin Wu, Yang Gao

**Affiliations:** 1 Graduate School, Tianjin Medical University, Tianjin, 300070 China; 2 Department of Imaging Diagnosis, Affiliated Hospital of Inner Mongolia Medical University, Hohhot, 010000 China; 3 GE Healthcare Co., Ltd., Shanghai, 210000 China; 4 Department of Radiology, Affiliated Zhongshan Hospital of Dalian University, Dalian, 116001 China

**Keywords:** Glioma, Histopathology, Radiomics, Magnetic resonance imaging

## Abstract

**Background:**

Genotype status of glioma have important significance to clinical treatment and prognosis. At present, there are few studies on the prediction of multiple genotype status in glioma by method of multi-sequence radiomics. The purpose of the study is to compare the performance of clinical features (age, sex, WHO grade, MRI morphological features etc.), radiomics features from multi MR sequence (T2WI, T1WI, DWI, ADC, CE-MRI (contrast enhancement)), and a combined multiple features model in predicting biomarker status (IDH, MGMT, TERT, 1p/19q of glioma.

**Methods:**

In this retrospective analysis, 81 glioma patients confirmed by histology were enrolled in this study. Five MRI sequences were used for radiomic feature extraction. Finally, 107 features were extracted from each sequence on Pyradiomics software, separately. These included 18 first-order metrics, such as the mean, standard deviation, skewness, and kurtosis etc., 14 shape features and second-order metrics including 24 grey level run length matrix (GLCM), 16 grey level run length matrix (GLRLM), 16 grey level size zone matrix (GLSZM), 5 neighboring gray tone difference matrix (NGTDM), and 14 grey level dependence matrix (GLDM). Then, Univariate analysis and LASSO (Least absolute shrinkage and selection operator regression model were used to data dimension reduction, feature selection, and radiomics signature building. Significant features (*p* < 0.05 by multivariate logistic regression were retained to establish clinical model, T1WI model, T2WI model, T1 + C (T1WI contrast enhancement model, DWI model and ADC model, multi sequence model. Clinical features were combined with multi sequence model to establish a combined model. The predictive performance was validated by receiver operating characteristic curve (ROC analysis and decision curve analysis (DCA).

**Results:**

The combined model showed the better performance in some groups of genotype status among some models (IDH AUC = 0.93, MGMT AUC = 0.88, TERT AUC = 0.76). Multi sequence model performed better than single sequence model in IDH, MGMT, TERT. There was no significant difference among the models in predicting 1p/19q status. Decision curve analysis showed combined model has higher clinical benefit than multi sequence model.

**Conclusion:**

Multi sequence model is an effective method to identify the genotype status of cerebral glioma. Combined with clinical models can better distinguish genotype status of glioma.

**Key Points:**

- The combined model showed the higher performance compare with other models in predicting genotype status of IDH, MGMT, TERT.
- Multi sequence model showed a better predictive model than that of a single sequence model.
- Compared with other models, the combined model and multi sequence model show no advantage in prediction of 1p/19q status.

**Supplementary Information:**

The online version contains supplementary material available at 10.1186/s12880-022-00865-8.

## Introduction

Gliomas are the most common primary neoplasms in brain, which represent approximately 30% of all central nervous system (CNS tumors and 80% of all malignant brain tumors [1]. Malignant glioma often develop in the higher ages, the high incidence being mostly within the 5th and 6th decades of life. Male and female incidence ratio of glioma is reported 1.47:1 [2]. Despite many advances have obtained in recent years, the average survival time still ranges from 17 weeks to 3 years [3]. The genotype status of glioma have been identified helpful recent years. The prognosis and survival rate of glioma is different with different genotype status. Accurate predicting the genotype status of glioma pre-operation can effectively predict prognosis and guide treatment strategy.

There have obtained many identifications about some phenotypes of glioma which are useful to prognosis, such as methylation of the MGMT, mutations in IDH1/2 and so on. More recently, co-deletion of 1q/19q and mutation in TERT is also important in prognosis and treatment option of glioma. However, there are little literature about radiomics combined with clinical characteristics to predict status of multiple phenotypes.

Isocitrate dehydrogenase (IDH catalyzes oxidative decarboxylation of isocitrate and plays an irreplaceable role in the Krebs cycle and cell homeostasis. IDH gene mutations occur in a variety of malignancies, including glioma, acute myeloid leukemia, cholangiocarcinoma, chondrosarcoma and thyroid cancer [4, 5]. Among gliomas, IDH mutations were found in 80% of WHO grade II/III gliomas [6]. A follow-up investigation showed that IDH mutation patients had a better prognosis. The median survival of GBM was 15 months in IDH wild type versus 31 months in IDH mutants. While anaplastic astrocytomas was 20 months in IDH wild type versus 65 months in IDH mutants [6]. Therefore, it is essential to predict IDH mutation status before treatment selection and patient stratification [7].

Human O6-methylguanine DNA methyltransferase (MGMT), also known as O6-alkylguanine DNA alkyltransferase (AGT), is a simple DNA repair protein involved in protecting the genome of normal cells from the mutagenicity of alkylating agents. The anti-mutation function of MGMT can block the cytotoxic effect of anticancer alkylation agents and make tumors resistant to chemotherapy drugs. When the MGMT promoter is methylated, the epigenetic expression of MGMT is inactivated and its resistance to alkylation agents lost, which manifested sensitive to chemotherapy. It will achieve better chemotherapy response, greater overall survival rate and longer progression time. Therefore, the detection of methylation status of MGMT promoter has become an important clinical procedure for the prognosis of glioma patients [8].

1p/19q co-deletion is that both the short arm of chromosome 1 (1p and the long arm of chromosome 19 (19q are deleted [9]. Lower-grade gliomas with both an IDH mutation (i.e., a mutation in either IDH1 or IDH2 and deletion of chromosome arms 1p and 19q (1p/19q codeletion), which occurs most often in oligodendrogliomas, have better responses to radiochemotherapy and are associated with longer survival than diffuse gliomas without these alterations [10]. A research showed that patients without chromosome 1p/19q co-deletions was associated with poor overall and progression-free survival [11].

Telomerase reverse transcriptase (TERT is important for the biology of diffuse gliomas. When DNA is replicated during mitosis, telomeres shorten, leading to apoptosis. Telomerase is an RNA-dependent DNA polymerase, which can prolong telomere DNA and maintain telomere homeostasis, leading to cell immortalization and the occurrence of malignant tumors. TRET is the rate-limiting catalytic subunit of telomerase. TERT promoter mutation can cause TERT transcription upregulation and activate telomerase [12]. TERT promoter mutations have been found in central nervous system tumors, including primary glioma (up to 80%), medulloblastoma (20%), and meningioma (6.4–11%) [13]. The frequency of TERT promoter mutations in gliomas increases with the increasing WHO grade. TERT promoter mutations were independently associated with poorer overall survival in all glioma subtypes [14].

Radiomics predicting prognosis of glioma pre-operation noninvasively is invaluable as biopsy is invasively and test of phenotypes is difficult available. Thus, radiomics can bringing huge benefits to patients who just not complete the test of phenotypes and to guide clinical pathway of treatment. So far, it has been confirmed that many molecular biomarkers are related to the prognosis of glioma, including IDH, MGMT, TERT, 1p/19q, EGFR, TP53, BRAF etc. In this study, we aimed to explore the valuable of radiomics in conventional (T2W/T1W/T1W + C and diffusion (DWI/ADC MR combined with clinical features (clinical and MRI imaging characteristics to predict four kinds of glioma molecular biomarkers (IDH, MGMT, TERT, 1p/19q).

## Material and methods

### Patient data

**Fig. 1:**
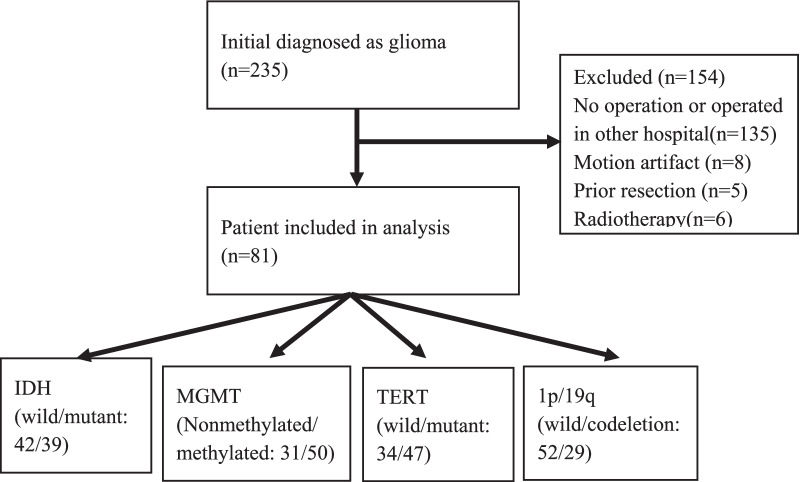
Flowchart of study population

Patients who were initial diagnosed with glioma between January 2018 and September 2021 were collected. A total of 235 patients with initial diagnosed as glioma were reviewed (Fig. 1). Inclusion criteria were (1 Glioma confirmed by surgical pathology and containing all genetic diagnostic information (IDH1, MGMT, TREF, and 1q/19q and (2 MRI scan performed within 30 days before surgery and including four sequences (T2W, T1W, T1W + C and DWI). Exclusion criteria were no surgery or surgery in another hospital (n = 135), poor MR image quality (motion artifact, n = 8), prior surgery (n = 5 or radiotherapy (n = 6). Finally, 81 patients met the criteria, including infiltrative and circumscribed gliomas (Table1). Our study complied with the Declaration of Helsinki and informed consent was waived due to the retrospective nature of the study by ethical committee of Affiliated Hospital of Inner Mongolia Medical University. All methods were carried out in accordance with relevant guidelines and regulations.

**Table 1:** Demographic data for each glioma biomarker

Total	Status	N (%)	Age (Q1, Q3)	Sex (M/F)	Enhancement (%)	WHO grade (I/II/III/IV)
		81 (100)		40/41	61 (75)	2/26/29/24
IDH						
	Wild-type	42 (52)	57 (44, 64)	21/21	38 (90)	2/9/10/21
	Mutant	39 (48)	44 (38, 51)	19/20	22 (56)	0/17/19/3
MGMT						
	Nonmethylated	31 (38)	57 (42, 64)	16/15	27 (87)	2/9/8/12
	Methylated	50 (62)	45 (39, 58)	24/26	33 (66)	0/17/21/12
TERT						
	Wild-type	34 (42)	46 (37, 57)	16/18	22 (65)	2/15/10/7
	Mutant	47 (58)	52 (43, 64)	24/23	38 (81)	0/11/19/17
1p/19q						
	Wild-type	52 (64)	53 (39, 62)	26/26	39 (75)	2/17/15/18
	codeletion	29 (36)	45 (42, 58)	14/15	21 (72)	0/9/14/6

Tissue samples were obtained from patients undergoing resection. Then sample section was used to perform diagnosis of neuropathology and molecular evaluation. Multiplex PCR combined with next generation sequencing were used to detect IDH1/2, TERT and 1q/19q co-deletion status. Pyrosequencing of bisulfite-treated genomic DNA (CpG sites 74–78, QIAGEN was used to detect MGMT promoter methylation status.

MR imaging was obtained using 2 MRI scanners (Skyra 3 T from Siemens Healthineers, Germany and Discovery 3 T from GE, Healthcare within our radiology department. The sequences and parameters of MR scan including: T2W (TR/TE, 5000–8000/100–200 ms, voxel size: 0.5 × 0.5 × 1 mm^3^), T1W (TR/TE, 1000–1800/10-25 ms, voxel size: 0.5 × 0.5 × 1 mm^3^), DWI (TR/TE: 3000–3800/66–81 ms, with *b* values of 0 and 1000 s/mm^2^, voxel size: 0.9 × 0.9 × 5.0 mm^3^ and post-contrast T1W imaging (TR/TE, 1000–1800/10–25 ms, voxel size: 0.5 × 0.5 × 1 mm^3^). A total volume of 0.1 mmol/kg of gadobutrol (Gadovist, Bayer Healthcare was injected intravenously for post-contrast T1W imaging.

### Clinical and MRI imaging data

Clinical features: patient sex, age, WHO grade (I, II, III, IV), genotype status of molecular biomarkers (IDH wild or mutant, MGMT nonmethylated or methylated, TERT wild or mutant, 1p/19q wild or codeletion (Table1).

MRI imaging features: tumour size (< 6 or > 6 cm), tumour centre location (left side or right side), cerebral lobe involving (frontal lobe, occipital lobe, parietal lobe, temporal lobe, insular lobe), cortex matter involving (involving or not), deep white matter involving (involving or not), pial matter involving (involving or not), ependymal membrane involving (involving or not), tumour cross midline (cross or not), oedema cross midline (cross or not), tumour border (clear or not), haemorrhage (yes or no), cystic_necrosis (no, < 25%, 25–50%, > 50%), oedema degree (< 1.6 cm or > 1.6 cm), enhancemengt_style (no, ring-enhancement, nodular-enhancement, irregular-enhancement), enhancement_degree (no, slight, obvious), signal characteristics (homogenous, heterogenous) [3].

### Models establishment

*Clinical model* Univariate analysis was used for clinical and imaging features, and the features with *p* < 0.05 were retained. Then multivariate stepwise regression was used to retain the smallest AIC (Akaike Information Criterion feature set. Finally, multivariate logistic regression was used to establish a multivariate clinical model.

*Radiomics model* For each patient, tumor segmentation was delineated manually slice by slice on T2WI by a 6 years of experience radiologist and under supervision of a 10 years of experience neuroradiologist. They were blinded to the final diagnosis and molecular biomarkers status. T1WI, T2WI, DWI, ADC and T1WI + C were co-registered after interscan motion corrections. The volume of interest (VOI was generated encompassing the entire region of T2WI hyperintensity by segmenting the region of interest (ROI slice by slice on axial scans. After additional reviewing session, any discrepancies in ROI were resolved by consensus. The VOI overlaid onto co-registered datasets, which obtained five VOI from each sequence. All segmentations and registrations were carried out by ITK-SNAP software (version 3.8.0, https://www.itksnap.org).

**Fig. 2:**
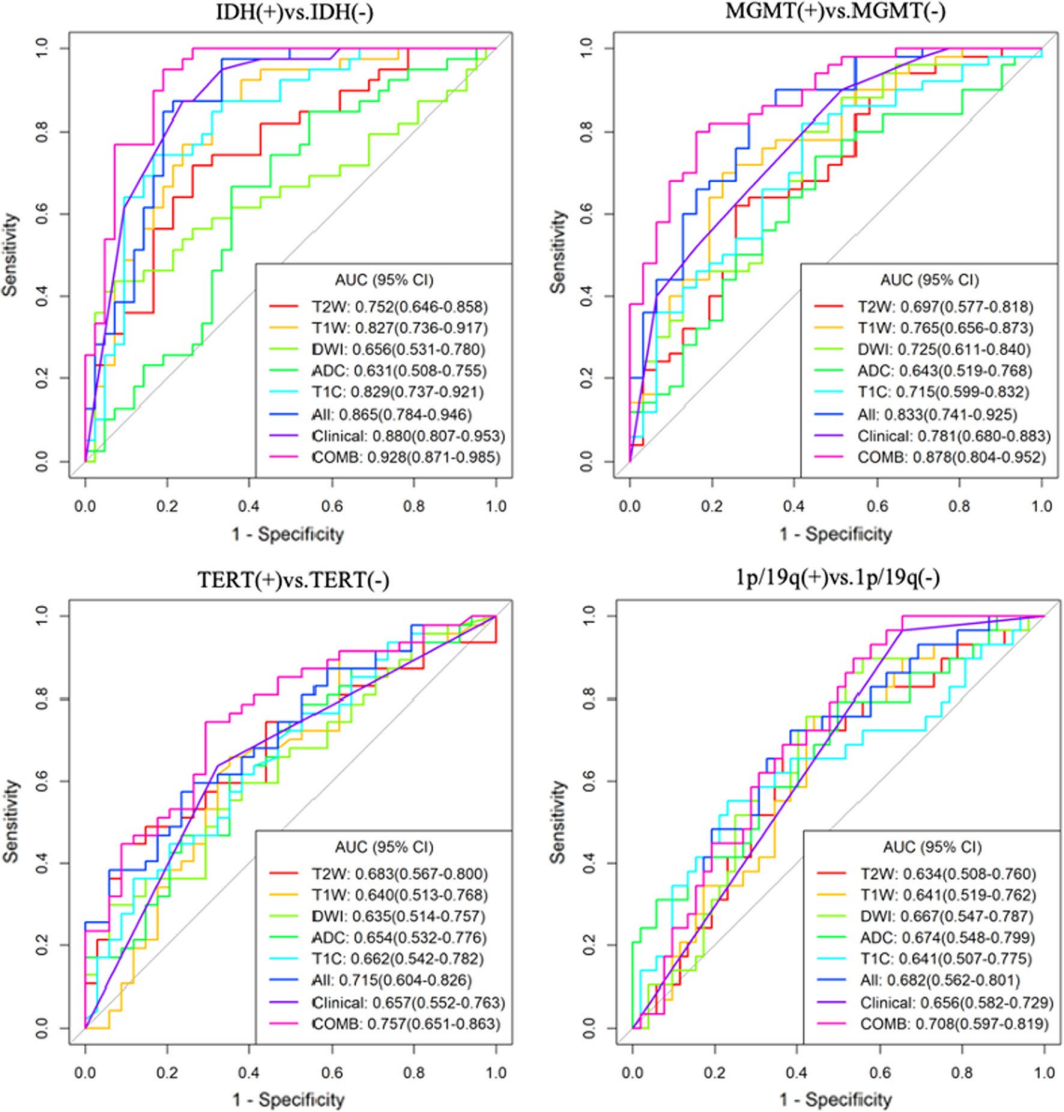
Receiver-operating characteristic (ROC curves for prediction of each biomarker status. combined model which unites clinical and radiomics features shows significant improvement in predicting each biomarker status, especially in group of IDH (0.928 and MGMT (0.878). T2W, T2WI model. T1W, T1WI model. DWI, DWI model. ADC, ADC model. T1C, T1 + C model. All, multi sequence model. Clinical, clinical model. COMB, combined model

Image processing and radiomics features extraction was performed on PHIgo-AK software (GE Healthcare, China which integrate Pyradiomics toolkit [15]. Automatic preprocessing was standardized for each case involving resampling (set vovel for 1 × 1 × 1mm to reduce the influence between different layer thicknesses and adopt linear interpolation), intensity normalization (z-score)and discretization (set binwidth to 5). The above VOI corresponding to each sequence is input into the corresponding image in the software of Pyradiomics toolkit to extract the radiomics features. The workflow is presented in Additional file 1: Fig. S2. At last, each sequence can extracted a total of 107 radiomic features. These included 18 first-order metrics, such as the mean, standard deviation, skewness, and kurtosis, 14 shape features and second-order metrics including 24 grey level run length matrix (GLCM), 16 grey level run length matrix (GLRLM), 16 grey level size zone matrix (GLSZM), 5 neighboring gray tone difference matrix (NGTDM), and 14 grey level dependence matrix (GLDM). The radiomic features selection procedure were performed in the cross-validation stage as follow: (1 Using univariate rank sum test, the characteristics of *p* < 0.05 were retained (2 Correlation analysis was used to remove features with a correlation greater than 0.8; (3 Least Absolute Shrinkage and Selection Operator (LASSO [16] regularization was employed to remove collinear features; (4 Multivariate stepwise regression was used to retain the feature set of minimal AIC. Each sequence is screened in this way.The independent radiomic model of each sequence radiomic features were established by multivariate logistic regression, including T1WI model, T2WI model, T1 + C model, DWI model and ADC model. Meaningful features from five sequences were combined to establish a combined radiomics model called multi sequence model (It is named ‘All’ model in the Figs. 2, 5).

*Combined model* The combine model was established by combining the radscore output from multi sequence model with the clinical characteristics by multivariate logistic regression.

All the above methods and models are completed separately for each group of molecular biomarkers.

### Statistical analysis

Statistical analysis was performed using R (version:4.1.0, https://www.rproject.org). A two-sided *p* value of less than 0.05 was considered statistically significant. Differences in the clinical and imaging characteristics between group of each genic phenotype were evaluated using the independent samples t test, the Mann–Whitney U test, and the chi-squared or Fisher’s exact tests, as appropriate. All models which include T2WI, T1WI, T1 + C, DWI, ADC, All, clinical, combined were established by logistic regression. Three-fold cross-validation repeated 20 times strategy was used to select best features subset. The cross-validation voting strategy [17], features selected in more than onefold were used as the final feature set. Receiver-operating characteristic (ROC curves and area under the curve (AUC was obtained. A comparative analysis between ROCs of all models in each group was performed using nonparametric methods described by DeLong et al. [18]. Decision curve analysis (DCA was carried out to assess the clinical usefulness of each model by evaluating the net benefit at various threshold probabilities.

## Results

### Clinical model assessment

Comparison of clinical and MRI imaging features of phenotype status for each molecular biomarker are summarized in Table 2 (Only the features with significant differences are listed, all features are detailed in Additional file 1: Table S1). After univariate analysis and multivariate stepwise regression, the differences in the following characteristics were statistically significant. Nine characteristics are predicting factors of IDH status including Age, Grade, Frontal lobe, Parietal lobe, Involving cortex matter, Oedema degree, Enhancement style, Enhancement degree and Signal characteristics. Five characteristics are predicting factors of MGMT status including Frontal lobe, Involving cortex matter, Border, Oedema degree and Enhancement degree. Enhancement style is predicting factor of 1p/19q status. Involving pial matter is predicting factor of TERT status. Above characteristics set were further selected to perform multivariate logistic regression analysis. The results are shown in Table 3. IDH mutant type is easier involving frontal lobe than wild type. IDH wild type is more common showed ring or irregular enhancement than mutant type. MGMT Methylated type is more likely involve frontal lobe and have a non-clear border than Nonmethylated type. Oedema degree of MGMT Methylated type is less than Nonmethylated type. TERT mutant type is more likely involving pial matter than wild type. Ring-enhancement is more likely presenting in 1p/19q wild type than co-deletion type. Then, a clinical model of each biomarker was established based on the independent variables mentioned above. IDH: the AUC was 0.88 (95% confidence interval, 0.81–0.95); MGMT: the AUC was 0.78 (95% confidence interval, 0.68–0.88); TERT: the AUC was 0.66 (95% confidence interval, 0.55–0.76; 1p/19q: the AUC was 0.66 (95% confidence interval, 0.58–0.73 (Fig. 2).

**Table 2:** Comparison of clinical and MRI imaging features of phenotype status for each molecular biomarker

Phenotypes	IDH			MGMT			TERT			1p/19q		
Stutas	Wild-type	Mutant	*p*-value	Nonmethylated	Methylated	*p*-value	Wild-type	Mutant	*p*-value	Wild-type	codeletion	*p*-value
N	42	39		31	50		34	47		52	29	
Age	57.0 [43.8;64.5]	44.0 [38.0;51.5]	0.003	57.0 [42.5;64.5]	45.5 [39.0;57.8]	0.054	46.5 [37.0;57.0]	52.0 [43.0;63.5]	0.074	53.0 [39.0;62.2]	45.0 [42.0;58.0]	0.294
Grade:			< 0.001			0.11			0.046			0.269
Grade I	2 (4.76%)	0 (0.00%)		2 (6.45%)	0 (0.00%)		2 (5.88%)	0 (0.00%)		2 (3.85%)	0 (0.00%)	
Grade II	9 (21.4%)	17 (43.6%)		9 (29.0%)	17 (34.0%)		15 (44.1%)	11 (23.4%)		17 (32.7%)	9 (31.0%)	
Grade III	10 (23.8%)	19 (48.7%)		8 (25.8%)	21 (42.0%)		10 (29.4%)	19 (40.4%)		15 (28.8%)	14 (48.3%)	
Grade IV	21 (50.0%)	3 (7.69%)		12 (38.7%)	12 (24.0%)		7 (20.6%)	17 (36.2%)		18 (34.6%)	6 (20.7%)	
Frontal lobe:			< 0.001			0.007			0.777			0.163
Non-involving	22 (52.4%)	4 (10.3%)		16 (51.6%)	10 (20.0%)		12 (35.3%)	14 (29.8%)		20 (38.5%)	6 (20.7%)	
Involving	20 (47.6%)	35 (89.7%)		15 (48.4%)	40 (80.0%)		22 (64.7%)	33 (70.2%)		32 (61.5%)	23 (79.3%)	
Parietal lobe:			0.001			0.104			0.644			0.087
Non-involving	28 (66.7%)	38 (97.4%)		22 (71.0%)	44 (88.0%)		29 (85.3%)	37 (78.7%)		39 (75.0%)	27 (93.1%)	
Involving	14 (33.3%)	1 (2.56%)		9 (29.0%)	6 (12.0%)		5 (14.7%)	10 (21.3%)		13 (25.0%)	2 (6.90%)	
Involving cortex matter:			0.001			0.027			0.202			0.087
Non-involving	14 (33.3%)	1 (2.56%)		10 (32.3%)	5 (10.0%)		9 (26.5%)	6 (12.8%)		13 (25.0%)	2 (6.90%)	
Involving	28 (66.7%)	38 (97.4%)		21 (67.7%)	45 (90.0%)		25 (73.5%)	41 (87.2%)		39 (75.0%)	27 (93.1%)	
Involving pial matter:			0.22			0.316			0.01			0.399
Non-involving	24 (57.1%)	16 (41.0%)		18 (58.1%)	22 (44.0%)		23 (67.6%)	17 (36.2%)		28 (53.8%)	12 (41.4%)	
Involving	18 (42.9%)	23 (59.0%)		13 (41.9%)	28 (56.0%)		11 (32.4%)	30 (63.8%)		24 (46.2%)	17 (58.6%)	
Border:			0.056			0.007			1			0.163
clear	18 (42.9%)	8 (20.5%)		16 (51.6%)	10 (20.0%)		11 (32.4%)	15 (31.9%)		20 (38.5%)	6 (20.7%)	
Non-clear	24 (57.1%)	31 (79.5%)		15 (48.4%)	40 (80.0%)		23 (67.6%)	32 (68.1%)		32 (61.5%)	23 (79.3%)	
Oedema degree:			0.008			0.011			0.419			0.752
< 1.6 cm	18 (42.9%)	29 (74.4%)		12 (38.7%)	35 (70.0%)		22 (64.7%)	25 (53.2%)		29 (55.8%)	18 (62.1%)	
> 1.6 cm	24 (57.1%)	10 (25.6%)		19 (61.3%)	15 (30.0%)		12 (35.3%)	22 (46.8%)		23 (44.2%)	11 (37.9%)	
Enhancemengt_style:			< 0.001			0.04			0.196			0.013
No	4 (9.52%)	17 (43.6%)		4 (12.9%)	17 (34.0%)		12 (35.3%)	9 (19.1%)		13 (25.0%)	8 (27.6%)	
Ring-enhancement	18 (42.9%)	1 (2.56%)		12 (38.7%)	7 (14.0%)		5 (14.7%)	14 (29.8%)		18 (34.6%)	1 (3.45%)	
Nodular-enhancement	5 (11.9%)	11 (28.2%)		6 (19.4%)	10 (20.0%)		8 (23.5%)	8 (17.0%)		8 (15.4%)	8 (27.6%)	
Irregular-enhancement	15 (35.7%)	10 (25.6%)		9 (29.0%)	16 (32.0%)		9 (26.5%)	16 (34.0%)		13 (25.0%)	12 (41.4%)	
Enhancement_degree:			< 0.001			0.006			0.202			0.095
No	4 (9.52%)	17 (43.5%)		4(12.9%)	17 (34.0%)		12 (35.3%)	9 (19.1%)		13 (25.0%)	8 (27.6%)	
Slight	4 (9.52%)	11 (28.2%)		3 (9.67%)	12 (24.0%)		7 (20.5%)	8 (17.0%)		6 (11.5%)	9 (31.0%)	
Obvious	34 (81.0%)	11 (28.2%)		24 (77.4%)	21 (42.0%)		15 (44.1%)	30 (63.8%)		33 (63.5%)	12 (41.4%)	
Signal characteristics:			0.038			0.19			1			1
Homogenous	2 (4.76%)	9 (23.1%)		2 (6.45%)	9 (18.0%)		5 (14.7%)	6 (12.8%)		7 (13.5%)	4 (13.8%)	
Heterogenous	40 (95.2%)	30 (76.9%)		29 (93.5%)	41 (82.0%)		29 (85.3%)	41 (87.2%)		45 (86.5%)	25 (86.2%)

**Table 3:** Multivariate logistic regression analysis of clinical characteristics for each Biomarker

Clinical characteristics	OR	95% CI	*p* value
*IDH*			
Frontal lobe (ref. non-involving)	8.043	2.057–38.67	0.004
Involving cortex matter (ref. non-involving)	7.395	0.879–167.248	0.105
Ring-enhancement (ref. no enhancement)	0.026	0.001–0.185	0.002
Irregular-enhancement (ref. no enhancement)	0.183	0.046–0.629	0.009
*MGMT*			
Frontal lobe (ref. non-involving)	5.262	1.674–18.673	0.006
Border (ref. clear)	4.031	1.317–13.326	0.017
Oedema degree(ref. < 1.6 cm)	0.184	0.054–0.546	0.004
*TERT*			
Involving pial matter (ref. non-involving)	3.690	1.481–9.664	0.006
*1p/19q*			
Ring-enhancement (ref. no enhancement)	0.067	0.004–0.359	0.011

### Radiomics model assessment

Following LASSO regularization and logistic regression analysis, multi sequence model was established. As a result, a total of 4 in IDH, 5 in MGMT, 4 in TERT, 2 in 1p/19q radiomic features (Additional file 1: Table S2; Fig. S3 remained as contributors in our predictive model with resultant AUC of 0.87 in IDH, 0.83 in MGMT, 0.72 in TERT, 0.68 in 1p/19q (Fig. 2). In different genotypes, the AUC of predictive performance of the model established according to each sequence was (IDH: 0.75 in T2W, 0.83 in T1W, 0.66 in DWI, 0.63 ADC, 0.83 in T1 + C; MGMT: 0.70 in T2W, 0.77 in T1W, 0.73 in DWI, 0.64 ADC, 0.72 in T1 + C; TERT: 0.68 in T2W, 0.64 in T1W, 0.64 in DWI, 0.65 ADC, 0.66 in T1 + C; 1p/19q: 0.63 in T2W, 0.64 in T1W, 0.66 in DWI, 0.67 ADC, 0.64 in T1 + C (Fig. 2).

### Model comparisons

**Fig. 3:**
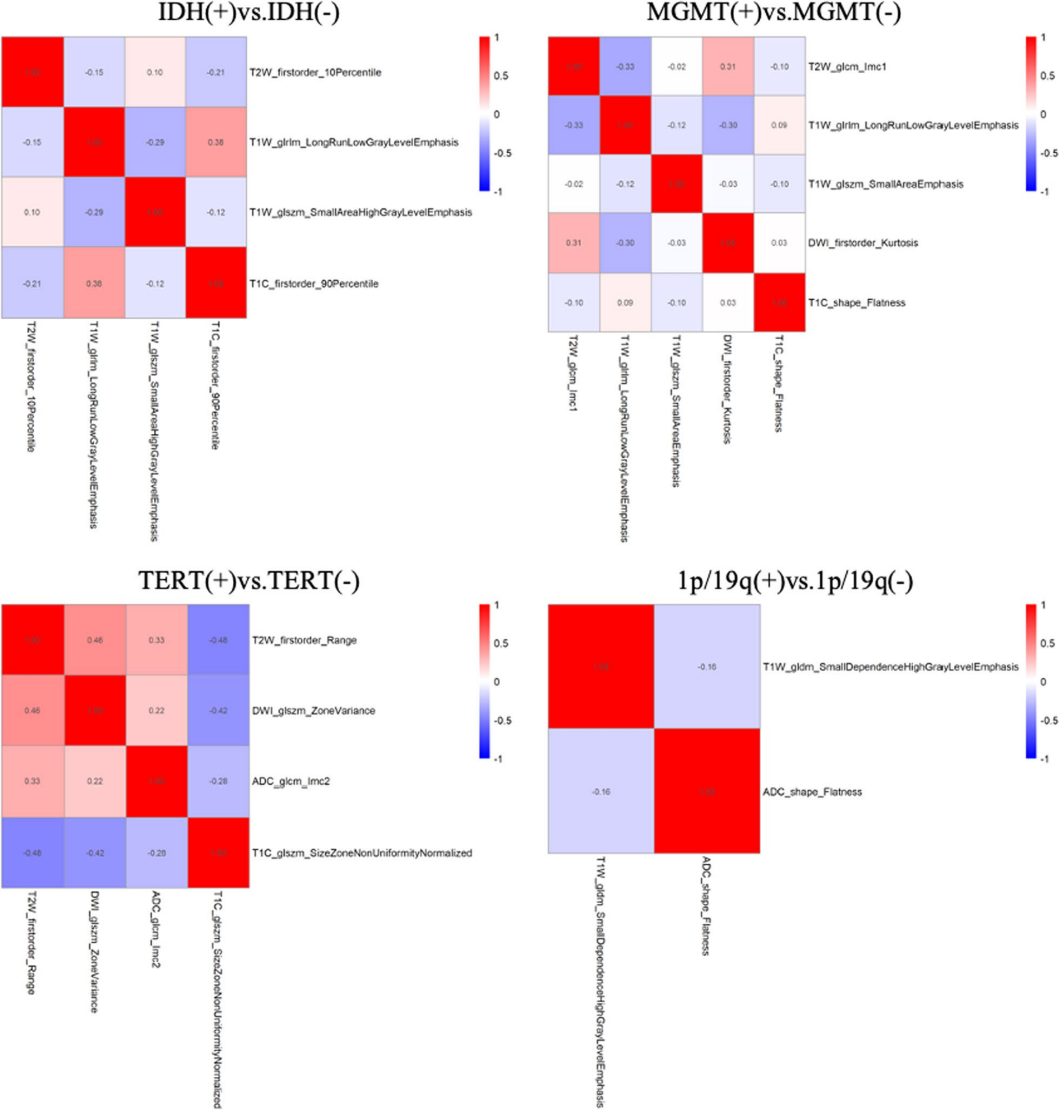
Heatmap comparison of the radiomic features which have significant statistic difference in each phenotypes group

**Fig. 4:**
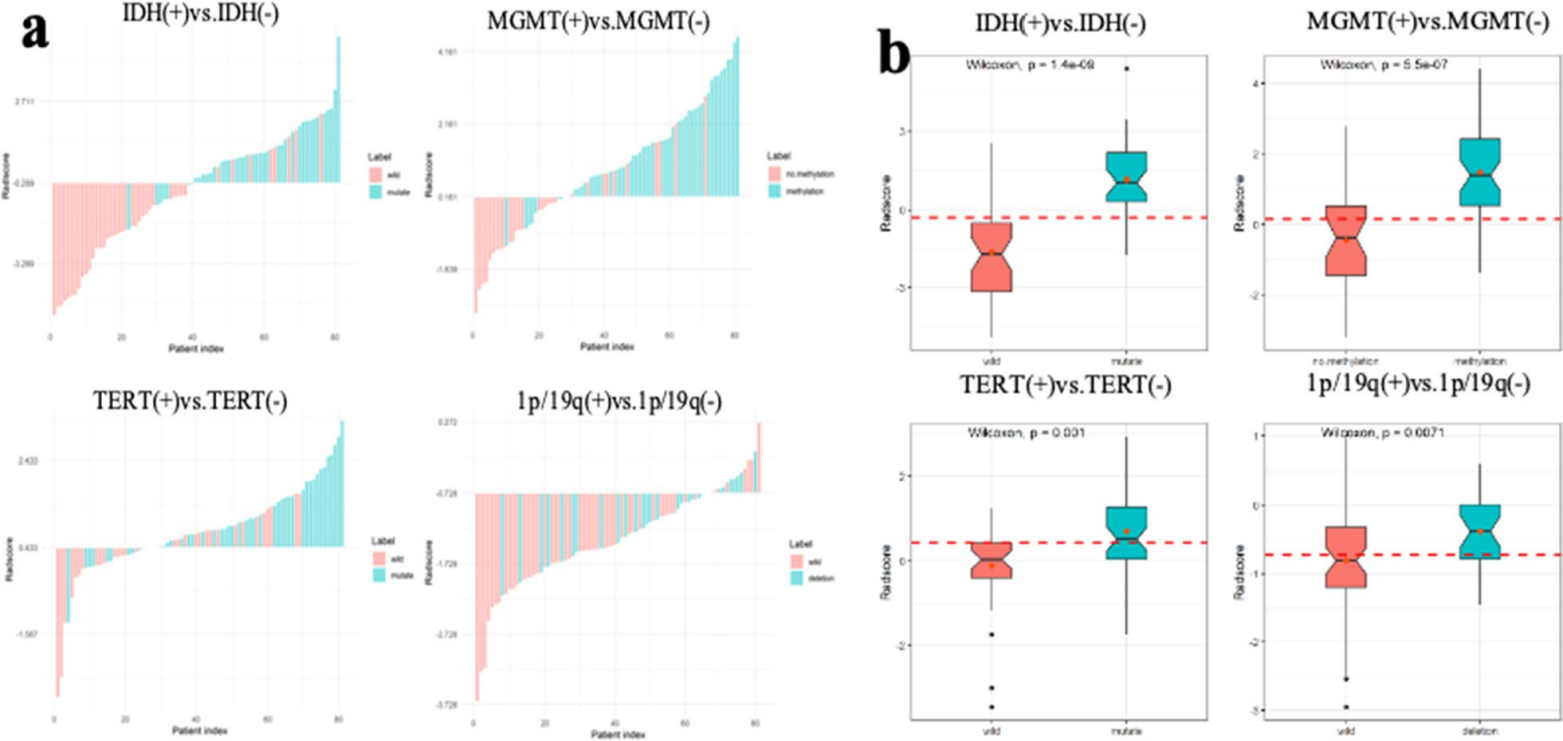
The bar (**a**) and box (**b**) chart of Radscore. The two charts showed a better performance in predicting each biomarker status, especially in group of IDH and MGMT

**Fig. 5:**
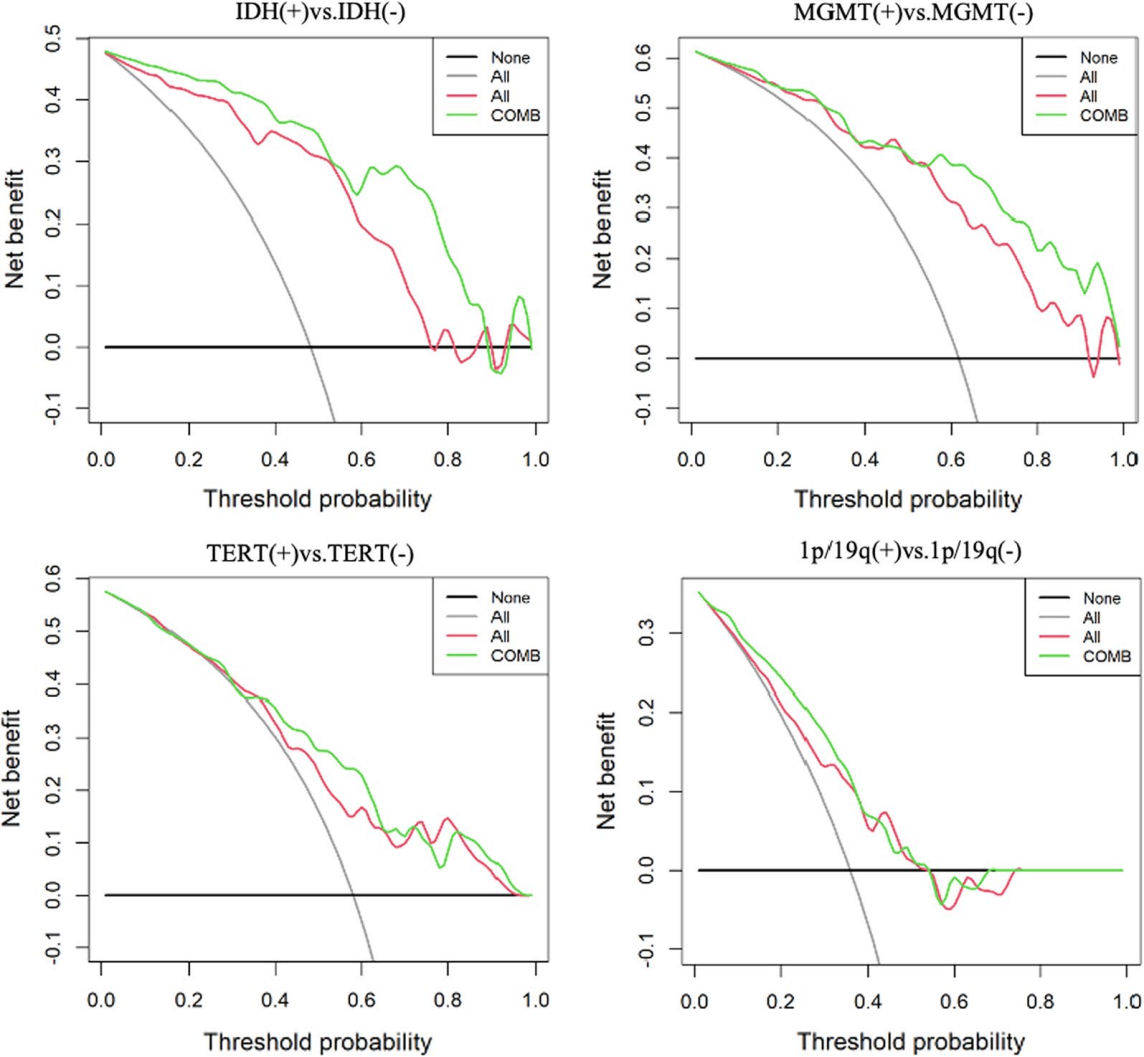
The decision curve analysis for multi sequence model (All, red curve and combined model (COMB, green curve). The Y-axis represents the net benefit

Nonparametric tests of DeLong were carried out between AUC of each model in each genotype. In IDH genotype, the AUC of multi sequence model is higher than that of T2WI model, DWI model and ADC model (All vs. T2W, DWI, ADC: 0.865 vs. 0.752, 0.656, 0.631, *p* < 0.05). The AUC of combined model is higher than that of T2WI model, T1WI model, T1 + C model and “All” model (COMB vs. T2W, T1W, T1C, All: 0.928 vs. 0.752, 0.827, 0.829, 0.865, *p* < 0.05). There was no significant difference in AUC between COMB model and clinical model (COMB vs. clinical: 0.928 vs. 0.880, *p* > 0.05). In MGMT genotype, the AUC of multi sequence model is higher than that of each single sequence model (All vs. T2W, T1W, DWI, ADC, T1 + C: 0.833 vs. 0.697, 0.765, 0.725, 0.643, 0.715, *p* < 0.05). The AUC of combined model is higher than that of T2WI model, T1WI model, DWI model, ADC model, T1 + C model and clinical model (COMB vs. T2W, T1W, DWI, ADC, T1C, clinical: 0.878 vs. 0.697, 0.765, 0.725, 0.643, 0.715, 0.781, *p* < 0.05). There was no significant difference in AUC between COMB model and “All” model (COMB vs. All: 0.878 vs. 0.833, *p* > 0.05). In TERT genotype, the AUC of multi sequence model is higher than that of DWI model and ADC model (All vs. DWI, ADC: 0.715 vs. 0.635, 0.654, *p* < 0.05). The AUC of combined model is higher than that of DWI model, ADC model and clinical model (COMB vs. DWI, ADC, clinical: 0.757 vs. 0.635, 0.654, 0.657, *p* < 0.05). In 1p/19q genotype, there is no significant difference in AUC value of each model. Detailed performances and ROC curves of all models are summarized in Fig. 2. A comparison of radiomic features which have significant predicting value in each biomarker is shown as a heat map in Fig. 3, respectively. Bar and box diagram according to radscore in each biomarker is shown in Fig. 4. Classification capability in IDH status group has a highest performance among all groups. DCA showed that the combined model has better predictive performance than the multi sequence radiomics model in each biomarker, and the best performance is in IDH group (Fig. 5).

## Discussion

Our results showed that constructed multiparametric model from MRI radiomics features can identify phenotype status of IDH, MGMT and TERT in preoperative MRI scans of patients with glioma. We specifically demonstrated that addition of clinical features can significantly improve predictive performance for IDH, MGMT, TERT.

There have had many literatures revealed the value of biomarkers status of glioma for diagnosis, therapy and prognosis. In this paper, four kinds of glioma biomarker were taken into consider to further investigate the value of predicting by radiomics add clinical model. The result showed radiomics model which include multiple parameters models obtained a higher predict performance (IDH AUC = 0.87, MGMT AUC = 0.83, TERT AUC = 0.72, 1p/19q AUC = 0.68 than each single parameter model in all kinds of phenotypes. These results are consistent with previous studies. A research showed an AUC of 0.884 and 0.669 for predicting IDH and TERT status, respectively [19]. Zhou et al. [20] demonstrated IDH and 1p/19q status prediction with AUC of 0.921 and 0.685 using a model from T1 + C and T2 FLAIR integrated with age. Kihira et al. [21] demonstrated IDH1 and MGMT status prediction with AUC of 1 and 0.79 using a combination model of texture features from conventional and diffusion.

IDH mutations are associated with increased survival in glioma patients [22] and increased sensitivity to temozolomide therapy [23] and radiation therapy [24]. LGG with IDH mutation along with 1p/19q codeletion has ever better clinical outcomes [10, 25]. MGMT promoter hypermethylation is the only known biomarker for TMZ response [26]. Mutation of TERT promoter as a genetic event is frequently detected in 60–75% of glioblastomas (GBM), and associated with a poor prognosis [27]. 1p/19q co-deletion is associated with better response to radiotherapy and alkylating agent chemotherapy, and longer progression-free and overall survival [28, 29].Therefore, preoperative identification of IDH, MGMT, TERT and 1p/19q status can play an important role with prognostic and treatment implications. Our results showing that IDH mutant type is easier involving frontal lobe than wild type. Feraco et al. demonstrated that IDH mutational status is more likely related to a frontal location [30]. IDH wild type is more common showed ring or irregular enhancement than mutant type, indicating a tendency for invasive behavior, which concurs with a study by Zhang et al. [31]. MGMT methylated type is more likely involve frontal lobe and have a non-clear border than Nonmethylated type, which are inconsistent with other studies. Previous studies suggested that MGMT promoter methylation is associated with GBM in parietal and occipital lobes [32]. These conflicting results may be need Multicenter studies further confirmed. Oedema degree of MGMT Methylated type is less than Nonmethylated type, indicating MGMT Methylated type is less aggressive, which is consistent with a research by Suh et al. [33]. In addition, our study also found that TERT mutant type is more likely involving pial matter than wild type and ring-enhancement is more likely presenting in 1p/19q wild type than co-deletion type. These results should be further confirmed by subsequent studies. Different genetic phenotypes status has different clinical imaging features. In-depth understanding of imaging features can better predict genetic phenotypes status of glioma. Establishing an accurate glioma biomarker prediction model integrate radiogenomics and clinical features will enable noninvasive prediction of prognosis and contributes to treatment planning.

Our study has several limitations. First, this is a retrospective study with a possible unknown bias. Second, as with other radiological studies, there is a risk of over-fitting given the large number of variables involved. Third, tumor segmentation is performed using the overall volume of the tumor with high signal on T2WI to increase inclusion. There was no differentiation of the cystic, edema and parenchyma areas. Further studies are needed to assess whether inclusion or exclusion of these components makes a significant difference in biomarker prediction. Finally, although the preprocessing uses techniques such as signal normalization and resampling to mitigate the impact of image changes caused by MR scanners from different vendors, external testing of the developed model through multi-institution collaboration is still required to improve the versatility and clinical utility of our model.

In conclusion, the described multi sequence model from all sequence radiomic features can better predict glioma biomarker status preoperatively. In particular, addition of clinical features provided significant added diagnostic value in determination of IDH, MGMT, TERT status.

## Supplementary Information

**Additional file 1.** The workflow shows the detailed process of radiomics feature extraction, including tumor segmentation, feature extraction, feature selection and signature analysis.

## Data Availability

The datasets used and/or analyzed during the current study are available from the corresponding author on reasonable request.

## Abbreviations

ADC: Apparent diffusion coefficient
DWI: Diffusion-weighted imaging
CE-MRI: Contrast enhancement
IDH: Isocitrate dehydrogenase
MGMT: Human O6-methylguanine DNA methyltransferase
TERT: Telomerase reverse transcriptase
GLCM: Gray-level cooccurrence matrix
GLDM: Gray-level dependence matrix
GLRLM: Gray-level run-length matrix
GLSZM: Gray-level size zone matrix
NGTDM: Neighboring gray tone difference matrix
LASSO: Least absolute shrinkage selection operator
ROC: Receiver-operating characteristic
AUC: Area under the curve
DCA: Decision curve analysis

## References

[1] Ostrom QT, Gittleman H, Liao P (2014). CBTRUS statistical report: primary brain and central nervous system tumors diagnosed in the United States in 2007–2011. Neuro Oncol..

[2] Pandith AA, Qasim I, Zahoor W (2018). Concordant association validates MGMT methylation and protein expression as favorable prognostic factors in glioma patients on alkylating chemotherapy (Temozolomide). Sci Rep.

[3] We J, Yang G, Hao X (2019). A multi-sequence and habitat-based MRI radiomics signature for preoperative prediction of MGMT promoter methylation in astrocytomas with prognostic implication. Eur Radiol.

[4] Berghoff AS, Kiesel B, Widhalm G (2017). Correlation of immune phenotype with IDH mutation in diffuse glioma. Neuro Oncol.

[5] Han S, Liu Y, Cai SJ (2020). IDH mutation in glioma: molecular mechanisms and potential therapeutic targets. Br J Cancer.

[6] Yan H, Parsons DW, Jin G (2009). IDH1 and IDH2 Mutations in Gliomas. N Engl J Med.

[7] Weller M, Bent M, Preusser M (2021). EANO guidelines on the diagnosis and treatment of diffuse gliomas of adulthood. Nat Rev Clin Oncol.

[8] Al-Obaide IMA, Arutla V (2021). Genomic space of MGMT in human glioma revisited: novel motifs, regulatory RNAs, NRF1, 2, and CTCF involvement in gene expression. Int J Mol Sci.

[9] Bent MJ, Brandes AA, Taphoorn MJB (2013). Adjuvant procarbazine, lomustine, and vincristine chemotherapy in newly diagnosed anaplastic oligodendroglioma: long-term follow-up of EORTC Brain Tumor Group Study 26951. J Clin Oncol.

[10] Brat DJ, Verhaak RGW, Dape KDA (2015). Comprehensive, integrative genomic analysis of diffuse lower-grade gliomas. N Engl J Med..

[11] Fan Z, Sun Z, Fang S (2021). Preoperative radiomics analysis of 1p/19q status in WHO grade II gliomas. Front Oncol.

[12] Patel B, Taiwo R, Kim AH (2020). TERT, a promoter of CNS malignancies. Neurooncol Adv..

[13] Killela PJ, Reitman ZJ, Jiao Y (2013). TERT promoter mutations occur frequently in gliomas and a subset of tumors derived from cells with low rates of self-renewal. Proc Natl Acad Sci USA.

[14] Heidenreich B, Rachakonda PS, Hosen I (2015). TERT promoter mutations and telomere length in adult malignant gliomas and recurrences. Oncotarget.

[15] van Griethuysen JJM, Fedorov A, Parmar C (2017). Computational radiomics system to decode the radiographic phenotype. Can Res.

[16] Tibshirani R (1996). Regression shriknage and selectino via the lasso. J R Stat Soc Ser B.

[17] Zhong Y, He J, Chalise P (2020). Nested and repeated cross validation for classification model with highdimensional data. Rev Colomb Estad.

[18] DeLong ER, DeLong DM, Clarke-Pearson DL (1988). Comparing the areas under two or more correlated receiver operating characteristic curves: a non-parametric approach. Biometrics.

[19] Yan J, Zhang B, Zhang S (2021). Quantitative MRI-based radiomics for noninvasively predicting molecular subtypes and survival in glioma patients. NPJ Precis Oncol..

[20] Zhou H, Chang K, Bai HX (2019). Machine learning reveals multimodal MRI patterns predictive of isocitrate dehydrogenase and 1p/19q status in diffuse lowand high-grade gliomas. J Neurooncol.

[21] Kihira S, Tsankova NM, Bauer A (2021). Multiparametric MRI texture analysis in prediction of glioma biomarker status: added value of MR diffusion. Neurooncol Adv..

[22] Huang LE, Cohen AL, Colman H (2017). IGFBP2 expression predicts IDH-mutant glioma patient survival. Oncotarget.

[23] Houillier C, Wang X, Kaloshi G (2010). IDH1 or IDH2 mutations predict longer survival and response to temozolomide in low-grade gliomas. Neurology.

[24] Li S, Chou AP, Chen W (2013). Overexpression of isocitrate dehydrogenase mutant proteins renders glioma cells more sensitive to radiation. Neuro Oncol.

[25] Hartmann C, Hentschel B, Simon M (2013). Long-term survival in primary glioblastoma with versus without isocitrate dehydrogenase mutations. Clin Cancer Res.

[26] Hegi ME, Diserens A-C, Gorlia T (2005). MGMT gene silencing and benefit from temozolomide in glioblastoma. N Engl J Med.

[27] Nonoguchi N, Ohta T, Oh J-E (2013). TERT promoter mutations in primary and secondary glioblastomas. Acta Neuropathol.

[28] Engelhard HH, Ana Stelea AM (2003). Oligodendroglioma and anaplastic oligodendroglioma: clinical features, treatment, and prognosis. Surg Neurol.

[29] Yao J, Hagiwara A, Raymond C (2020). Human IDH mutant 1p/19q co-deleted gliomas have low tumor acidity as evidenced by molecular MRI and PET: a retrospective study. Sci Rep..

[30] Feraco P, Bacci A, Ferrazza P (2020). Magnetic resonance imaging derived biomarkers of IDH mutation status and overall survival in grade III astrocytomas. Diagnostics.

[31] Zhang J, Peng H, Wang Y-L (2021). Predictive role of the apparent diffusion coefficient and MRI morphologic features on IDH status in patients with diffuse glioma: a retrospective cross-sectional study. Front Oncol.

[32] Eoli M, Menghi F, Bruzzone MG (2007). Methylation of O6-methylguanine DNA methyltransferase and loss of heterozygosity on 19q and/or 17p are overlapping features of secondary glioblastomas with prolonged survival. Clin Cancer Res.

[33] Suh CH, Kim HS, Jung SC (2018). Clinically relevant imaging features for MGMT promoter methylation in multiple glioblastoma studies: a systematic review and meta-analysis. AJNR Am J Neuroradiol.

