# Supplementary material for: Multiparametric MR radiomics in brain glioma: models comparation to predict biomarker status
Source: BMC Med Imaging. 2022 Aug 5;22:137. doi: 10.1186/s12880-022-00865-8 (PMC9354364; doi:10.1186/s12880-022-00865-8)

**Supplementary Data:**

**Supplementary Table 1** Comparison of clinical and MRI imaging features of phenotype status for each molecular biomarker

| Phenotypes | IDH | |  | MGMT | |  | TERT | |  | 1p/19q | |  |
| --- | --- | --- | --- | --- | --- | --- | --- | --- | --- | --- | --- | --- |
| Stutas | Wild-type | Mutant | p-value | Nonmethylated | Methylated | p-value | Wild-type | Mutant | p-value | Wild-type | codeletion | p-value |
| N | 42 | 39 |  | 31 | 50 |  | 34 | 47 |  | 52 | 29 |  |
| Gender: |  |  | 1 |  |  | 0.93 |  |  | 0.896 |  |  | 1 |
| female | 21 (50.0%) | 20 (51.3%) |  | 15 (48.4%) | 26 (52.0%) |  | 18 (52.9%) | 23 (48.9%) |  | 26 (50.0%) | 15 (51.7%) |  |
| male | 21 (50.0%) | 19 (48.7%) |  | 16 (51.6%) | 24 (48.0%) |  | 16 (47.1%) | 24 (51.1%) |  | 26 (50.0%) | 14 (48.3%) |  |
| Age | 57.0 [43.8;64.5] | 44.0 [38.0;51.5] | 0.003 | 57.0 [42.5;64.5] | 45.5 [39.0;57.8] | 0.054 | 46.5 [37.0;57.0] | 52.0 [43.0;63.5] | 0.074 | 53.0 [39.0;62.2] | 45.0 [42.0;58.0] | 0.294 |
| Grade: |  |  | <0.001 |  |  | 0.11 |  |  | 0.046 |  |  | 0.269 |
| Grade I | 2 (4.76%) | 0 (0.00%) |  | 2 (6.45%) | 0 (0.00%) |  | 2 (5.88%) | 0 (0.00%) |  | 2 (3.85%) | 0 (0.00%) |  |
| Grade II | 9 (21.4%) | 17 (43.6%) |  | 9 (29.0%) | 17 (34.0%) |  | 15 (44.1%) | 11 (23.4%) |  | 17 (32.7%) | 9 (31.0%) |  |
| Grade III | 10 (23.8%) | 19 (48.7%) |  | 8 (25.8%) | 21 (42.0%) |  | 10 (29.4%) | 19 (40.4%) |  | 15 (28.8%) | 14 (48.3%) |  |
| Grade IV | 21 (50.0%) | 3 (7.69%) |  | 12 (38.7%) | 12 (24.0%) |  | 7 (20.6%) | 17 (36.2%) |  | 18 (34.6%) | 6 (20.7%) |  |
| Gen IDH |  |  |  |  |  | <0.001 |  |  | 0.399 |  |  | <0.001 |
| Wild-type |  |  |  | 29 (93.5%) | 13 (26.0%) |  | 20 (58.8%) | 22 (46.8%) |  | 36 (69.2%) | 6 (20.7%) |  |
| Mutant |  |  |  | 2 (6.45%) | 37 (74.0%) |  | 14 (41.2%) | 25 (53.2%) |  | 16 (30.8%) | 23 (79.3%) |  |
| Gen_1p19q: |  |  | <0.001 |  |  | 0.002 |  |  | 0.028 |  |  |  |
| Wild-type | 36 (85.7%) | 16 (41.0%) |  | 27 (87.1%) | 25 (50.0%) |  | 27 (79.4%) | 25 (53.2%) |  |  |  |  |
| codeletion | 6 (14.3%) | 23 (59.0%) |  | 4 (12.9%) | 25 (50.0%) |  | 7 (20.6%) | 22 (46.8%) |  |  |  |  |
| Gen TERT: |  |  | 0.399 |  |  | 0.106 |  |  |  |  |  | 0.028 |
| Wild-type | 20 (47.6%) | 14 (35.9%) |  | 17 (54.8%) | 17 (34.0%) |  |  |  |  | 27 (51.9%) | 7 (24.1%) |  |
| Mutant | 22 (52.4%) | 25 (64.1%) |  | 14 (45.2%) | 33 (66.0%) |  |  |  |  | 25 (48.1%) | 22 (75.9%) |  |
| Gen MGMT: |  |  | <0.001 |  |  |  |  |  | 0.106 |  |  | 0.002 |
| Nonmethylated | 29 (69.0%) | 2 (5.13%) |  |  |  |  | 17 (50.0%) | 14 (29.8%) |  | 27 (51.9%) | 4 (13.8%) |  |
| methylated | 13 (31.0%) | 37 (94.9%) |  |  |  |  | 17 (50.0%) | 33 (70.2%) |  | 25 (48.1%) | 25 (86.2%) |  |
| Tumour size: |  |  | 0.993 |  |  | 0.559 |  |  | 0.553 |  |  | 0.817 |
| <6cm | 28 (66.7%) | 25 (64.1%) |  | 22 (71.0%) | 31 (62.0%) |  | 24 (70.6%) | 29 (61.7%) |  | 35 (67.3%) | 18 (62.1%) |  |
| >6cm | 14 (33.3%) | 14 (35.9%) |  | 9 (29.0%) | 19 (38.0%) |  | 10 (29.4%) | 18 (38.3%) |  | 17 (32.7%) | 11 (37.9%) |  |
| Tumour centre location: |  |  | 0.592 |  |  | 0.349 |  |  | 1 |  |  | 0.961 |
| Left-side | 24 (57.1%) | 19 (48.7%) |  | 19 (61.3%) | 24 (48.0%) |  | 18 (52.9%) | 25 (53.2%) |  | 27 (51.9%) | 16 (55.2%) |  |
| Right-side | 18 (42.9%) | 20 (51.3%) |  | 12 (38.7%) | 26 (52.0%) |  | 16 (47.1%) | 22 (46.8%) |  | 25 (48.1%) | 13 (44.8%) |  |
| Frontal lobe: |  |  | <0.001 |  |  | 0.007 |  |  | 0.777 |  |  | 0.163 |
| Non-involving | 22 (52.4%) | 4 (10.3%) |  | 16 (51.6%) | 10 (20.0%) |  | 12 (35.3%) | 14 (29.8%) |  | 20 (38.5%) | 6 (20.7%) |  |
| Involving | 20 (47.6%) | 35 (89.7%) |  | 15 (48.4%) | 40 (80.0%) |  | 22 (64.7%) | 33 (70.2%) |  | 32 (61.5%) | 23 (79.3%) |  |
| Occipital lobe: |  |  | 0.117 |  |  | 0.154 |  |  | 0.635 |  |  | 0.291 |
| Non-involving | 38 (90.5%) | 39 (100%) |  | 28 (90.3%) | 49 (98.0%) |  | 33 (97.1%) | 44 (93.6%) |  | 48 (92.3%) | 29 (100%) |  |
| Involving | 4 (9.52%) | 0 (0.00%) |  | 3 (9.68%) | 1 (2.00%) |  | 1 (2.94%) | 3 (6.38%) |  | 4 (7.69%) | 0 (0.00%) |  |
| Parietal lobe: |  |  | 0.001 |  |  | 0.104 |  |  | 0.644 |  |  | 0.087 |
| Non-involving | 28 (66.7%) | 38 (97.4%) |  | 22 (71.0%) | 44 (88.0%) |  | 29 (85.3%) | 37 (78.7%) |  | 39 (75.0%) | 27 (93.1%) |  |
| Involving | 14 (33.3%) | 1 (2.56%) |  | 9 (29.0%) | 6 (12.0%) |  | 5 (14.7%) | 10 (21.3%) |  | 13 (25.0%) | 2 (6.90%) |  |
| Temporal lobe: |  |  | 0.122 |  |  | 0.579 |  |  | 0.38 |  |  | 0.473 |
| Non-involving | 27 (64.3%) | 32 (82.1%) |  | 21 (67.7%) | 38 (76.0%) |  | 27 (79.4%) | 32 (68.1%) |  | 36 (69.2%) | 23 (79.3%) |  |
| Involving | 15 (35.7%) | 7 (17.9%) |  | 10 (32.3%) | 12 (24.0%) |  | 7 (20.6%) | 15 (31.9%) |  | 16 (30.8%) | 6 (20.7%) |  |
| Insular lobe: |  |  | 1 |  |  | 1 |  |  | 0.823 |  |  | 0.127 |
| Non-involving | 35 (83.3%) | 32 (82.1%) |  | 26 (83.9%) | 41 (82.0%) |  | 29 (85.3%) | 38 (80.9%) |  | 46 (88.5%) | 21 (72.4%) |  |
| Involving | 7 (16.7%) | 7 (17.9%) |  | 5 (16.1%) | 9 (18.0%) |  | 5 (14.7%) | 9 (19.1%) |  | 6 (11.5%) | 8 (27.6%) |  |
| Involving cortex matter: |  |  | 0.001 |  |  | 0.027 |  |  | 0.202 |  |  | 0.087 |
| Non-involving | 14 (33.3%) | 1 (2.56%) |  | 10 (32.3%) | 5 (10.0%) |  | 9 (26.5%) | 6 (12.8%) |  | 13 (25.0%) | 2 (6.90%) |  |
| Involving | 28 (66.7%) | 38 (97.4%) |  | 21 (67.7%) | 45 (90.0%) |  | 25 (73.5%) | 41 (87.2%) |  | 39 (75.0%) | 27 (93.1%) |  |
| Involving deep white matter: |  |  | 0.656 |  |  | 0.831 |  |  | 1 |  |  | 0.975 |
| Non-involving | 8 (19.0%) | 10 (25.6%) |  | 6 (19.4%) | 12 (24.0%) |  | 8 (23.5%) | 10 (21.3%) |  | 11 (21.2%) | 7 (24.1%) |  |
| Involving | 34 (81.0%) | 29 (74.4%) |  | 25 (80.6%) | 38 (76.0%) |  | 26 (76.5%) | 37 (78.7%) |  | 41 (78.8%) | 22 (75.9%) |  |
| Involving pial matter: |  |  | 0.22 |  |  | 0.316 |  |  | 0.01 |  |  | 0.399 |
| Non-involving | 24 (57.1%) | 16 (41.0%) |  | 18 (58.1%) | 22 (44.0%) |  | 23 (67.6%) | 17 (36.2%) |  | 28 (53.8%) | 12 (41.4%) |  |
| Involving | 18 (42.9%) | 23 (59.0%) |  | 13 (41.9%) | 28 (56.0%) |  | 11 (32.4%) | 30 (63.8%) |  | 24 (46.2%) | 17 (58.6%) |  |
| Involving  ependymal membrane: |  |  | 0.15 |  |  | 0.448 |  |  | 1 |  |  | 0.163 |
| Non-involving | 25 (59.5%) | 30 (76.9%) |  | 19 (61.3%) | 36 (72.0%) |  | 23 (67.6%) | 32 (68.1%) |  | 32 (61.5%) | 23 (79.3%) |  |
| Involving | 17 (40.5%) | 9 (23.1%) |  | 12 (38.7%) | 14 (28.0%) |  | 11 (32.4%) | 15 (31.9%) |  | 20 (38.5%) | 6 (20.7%) |  |
| Tumour cross midline: |  |  | 1 |  |  | 0.677 |  |  | 1 |  |  | 0.703 |
| Non-cross | 32 (76.2%) | 30 (76.9%) |  | 25 (80.6%) | 37 (74.0%) |  | 26 (76.5%) | 36 (76.6%) |  | 41 (78.8%) | 21 (72.4%) |  |
| cross | 10 (23.8%) | 9 (23.1%) |  | 6 (19.4%) | 13 (26.0%) |  | 8 (23.5%) | 11 (23.4%) |  | 11 (21.2%) | 8 (27.6%) |  |
| Oedema cross midline: |  |  | 0.481 |  |  | 0.186 |  |  | 1 |  |  | 0.604 |
| Non-cross | 33 (78.6%) | 27 (69.2%) |  | 26 (83.9%) | 34 (68.0%) |  | 25 (73.5%) | 35 (74.5%) |  | 40 (76.9%) | 20 (69.0%) |  |
| cross | 9 (21.4%) | 12 (30.8%) |  | 5 (16.1%) | 16 (32.0%) |  | 9 (26.5%) | 12 (25.5%) |  | 12 (23.1%) | 9 (31.0%) |  |
| Border: |  |  | 0.056 |  |  | 0.007 |  |  | 1 |  |  | 0.163 |
| clear | 18 (42.9%) | 8 (20.5%) |  | 16 (51.6%) | 10 (20.0%) |  | 11 (32.4%) | 15 (31.9%) |  | 20 (38.5%) | 6 (20.7%) |  |
| Non-clear | 24 (57.1%) | 31 (79.5%) |  | 15 (48.4%) | 40 (80.0%) |  | 23 (67.6%) | 32 (68.1%) |  | 32 (61.5%) | 23 (79.3%) |  |
| Haemorrhage: |  |  | 0.909 |  |  | 0.829 |  |  | 0.069 |  |  | 0.475 |
| Non-Haemorrhage | 33 (78.6%) | 32 (82.1%) |  | 24 (77.4%) | 41 (82.0%) |  | 31 (91.2%) | 34 (72.3%) |  | 40 (76.9%) | 25 (86.2%) |  |
| Haemorrhage | 9 (21.4%) | 7 (17.9%) |  | 7 (22.6%) | 9 (18.0%) |  | 3 (8.82%) | 13 (27.7%) |  | 12 (23.1%) | 4 (13.8%) |  |
| Cystic_necrosis: |  |  | 0.896 |  |  | 0.915 |  |  | 0.94 |  |  | 0.354 |
| No | 7 (16.7%) | 9 (23.1%) |  | 7 (22.6%) | 9 (18.0%) |  | 7 (20.6%) | 9 (19.1%) |  | 12 (23.1%) | 4 (13.8%) |  |
| <25% | 18 (42.9%) | 16 (41.0%) |  | 12 (38.7%) | 22 (44.0%) |  | 15 (44.1%) | 19 (40.4%) |  | 19 (36.5%) | 15 (51.7%) |  |
| 25-50% | 9 (21.4%) | 8 (20.5%) |  | 6 (19.4%) | 11 (22.0%) |  | 6 (17.6%) | 11 (23.4%) |  | 13 (25.0%) | 4 (13.8%) |  |
| >50% | 8 (19.0%) | 6 (15.4%) |  | 6 (19.4%) | 8 (16.0%) |  | 6 (17.6%) | 8 (17.0%) |  | 8 (15.4%) | 6 (20.7%) |  |
| Oedema degree: |  |  | 0.008 |  |  | 0.011 |  |  | 0.419 |  |  | 0.752 |
| <1.6cm | 18 (42.9%) | 29 (74.4%) |  | 12 (38.7%) | 35 (70.0%) |  | 22 (64.7%) | 25 (53.2%) |  | 29 (55.8%) | 18 (62.1%) |  |
| >1.6cm | 24 (57.1%) | 10 (25.6%) |  | 19 (61.3%) | 15 (30.0%) |  | 12 (35.3%) | 22 (46.8%) |  | 23 (44.2%) | 11 (37.9%) |  |
| Enhancemengt_style: |  |  | <0.001 |  |  | 0.04 |  |  | 0.196 |  |  | 0.013 |
| No | 4 (9.52%) | 17 (43.6%) |  | 4 (12.9%) | 17 (34.0%) |  | 12 (35.3%) | 9 (19.1%) |  | 13 (25.0%) | 8 (27.6%) |  |
| Ring-enhancement | 18 (42.9%) | 1 (2.56%) |  | 12 (38.7%) | 7 (14.0%) |  | 5 (14.7%) | 14 (29.8%) |  | 18 (34.6%) | 1 (3.45%) |  |
| Nodular-enhancement | 5 (11.9%) | 11 (28.2%) |  | 6 (19.4%) | 10 (20.0%) |  | 8 (23.5%) | 8 (17.0%) |  | 8 (15.4%) | 8 (27.6%) |  |
| Irregular-enhancement | 15 (35.7%) | 10 (25.6%) |  | 9 (29.0%) | 16 (32.0%) |  | 9 (26.5%) | 16 (34.0%) |  | 13 (25.0%) | 12 (41.4%) |  |
| Enhancement_degree: |  |  | <0.001 |  |  | 0.006 |  |  | 0.202 |  |  | 0.095 |
| No | 4 (9.52%) | 17 (43.5%) |  | 4(12.9%) | 17 (34.0%) |  | 12 (35.3%) | 9 (19.1%) |  | 13 (25.0%) | 8 (27.6%) |  |
| Slight | 4 (9.52%) | 11 (28.2%) |  | 3 (9.67%) | 12 (24.0%) |  | 7 (20.5%) | 8 (17.0%) |  | 6 (11.5%) | 9 (31.0%) |  |
| Obvious | 34 (81.0%) | 11 (28.2%) |  | 24 (77.4%) | 21 (42.0%) |  | 15 (44.1%) | 30 (63.8%) |  | 33 (63.5%) | 12 (41.4%) |  |
| Signal characteristics: |  |  | 0.038 |  |  | 0.19 |  |  | 1 |  |  | 1 |
| Homogenous | 2 (4.76%) | 9 (23.1%) |  | 2 (6.45%) | 9 (18.0%) |  | 5 (14.7%) | 6 (12.8%) |  | 7 (13.5%) | 4 (13.8%) |  |
| Heterogenous | 40 (95.2%) | 30 (76.9%) |  | 29 (93.5%) | 41 (82.0%) |  | 29 (85.3%) | 41 (87.2%) |  | 45 (86.5%) | 25 (86.2%) |  |

**Supplementary Table 2**:

List of contributing texture features from conventional MRI (T2WI, T1WI, DWI, ADC, T1c+) following LASSO regularization and logistic regression analysis

**IDH:**

T2WI – First Order 10 percentile

T1WI – GLRLM Long Run Low Gray Level Emphasis

T1WI – GLSZM Small Area High Gray Level Emphasis

T1c+ – First Order 90 percentile

**MGMT:**

T2WI – GLCM Imc1

T1WI – GLRLM Long Run Low Gray Level Emphasis

T1WI – GLSZM Small Area Emphasis

DWI – First Order Kurtosis

T1c+ – Shape Flatness

**TERT:**

T2WI – Firstorder_Range

DWI – GLSZM Zone Variance

ADC – GLCM Imc2

T1c+ – GLSZM Size Zone Non-Uniformity Normalized

**1p/19q:**

ADC – Shape Flatness

T1c+ – GLDM Small Dependence High Gray Level Emphasis

**Supplementary Figures:**

**
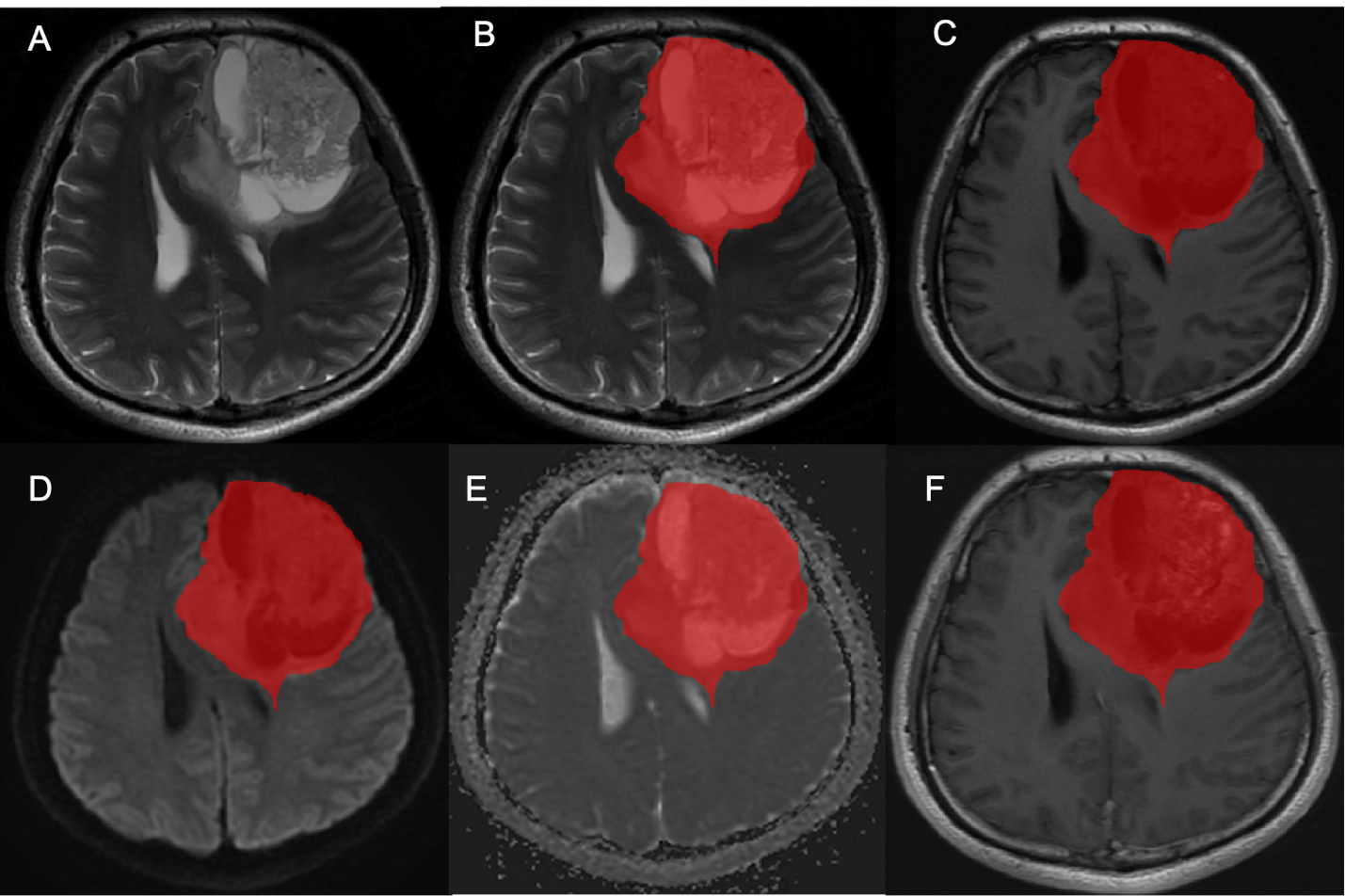
**

**Supplementary Figure 1.** Example of tumor segmentation. Axial images from a preoperative MRI are shown in a 36-year-old man with WHO grade III glioblastoma with biomarker profile of *IDH1* mutant, *MGMT* methylation, *TERT* wildtype, *1p/19q* wildtype. Using T2WI images (A), a volume of interest (VOI) was generated using a voxel-based signal intensity threshold method subsuming the entire region of T2WI hyperintensity (B). This VOI was then overlaid onto coregistered T1WI(C), diffusion-b1000 (D), ADC map (E) and T1c+ (F).

**Supplementary Figure 2** Radiomics signature workflow


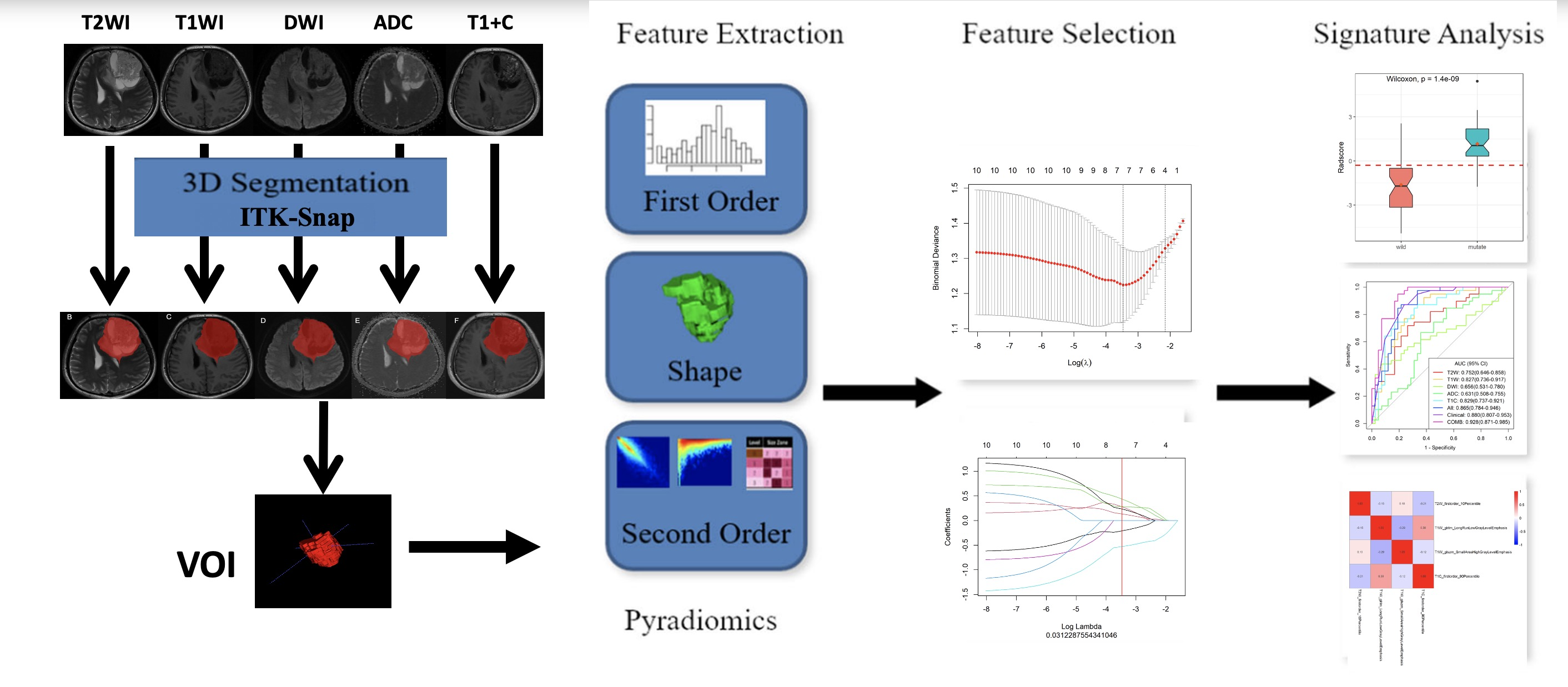

Supplement: Supplementary file 1 — Additional file 1. The workflow shows the detailed process of radiomics feature extraction, including tumor segmentation, feature extraction, feature selection and signature analysis. [file 12880_2022_865_MOESM1_ESM.docx]
